# An Automatic Segmentation Method for Lung Tumor Based on Improved Region Growing Algorithm

**DOI:** 10.3390/diagnostics12122971

**Published:** 2022-11-28

**Authors:** Monan Wang, Donghui Li

**Affiliations:** Mechanical & Power Engineering College, Harbin University of Science and Technology, Harbin 150080, China

**Keywords:** improved region growing algorithm, lung tumor segmentation, growth restriction conditions, automatically update thresholds

## Abstract

In medical image processing, accurate segmentation of lung tumors is very important. Computer-aided accurate segmentation can effectively assist doctors in surgery planning and treatment decisions. Although the accurate segmentation results of lung tumors can provide a reliable basis for clinical treatment, the key to obtaining accurate segmentation results is how to improve the segmentation performance of the algorithm. We propose an automatic segmentation method for lung tumors based on an improved region growing algorithm, which uses the prior information on lung tumors to achieve an automatic selection of the initial seed point. The proposed method includes a seed point expansion mechanism and an automatic threshold update mechanism and takes the combination of multiple segmentation results as the final segmentation result. In the lung image database consortium (LIDC-IDRI) dataset, we designed 10 experiments to test the proposed method and compare it with 4 popular segmentation methods. The experimental results show that the average dice coefficient obtained by the proposed method is 0.936 ± 0.027, and the average Jaccard distance is 0.114 ± 0.049. The average dice coefficient obtained by the proposed method is 0.107, 0.053, 0.040, and 0.156, higher than that of the other four methods, respectively. This study proves that the proposed method can automatically segment lung tumors in CT slices and has suitable segmentation performance.

## 1. Introduction

Lung cancer has always been a topic of great concern in the world, and it also accounts for nearly 20% of cancer mortality in the world [1]. The lung is an important organ of the human respiratory system, and lung tumor is also directly related to human health. Early surgical planning and treatment can improve the cure rate and increase the survival rate of patients [2,3]. Computer-aided accurate segmentation can effectively assist doctors in surgery planning and treatment decisions [4,5]. The purpose of lung tumor segmentation based on computed tomography (CT) images is to accurately segment lung tumors from CT images and then assist doctors in treatment planning. Therefore, it is of great significance to accurately segment lung tumors from CT images. The accurate segmentation results can also provide a reliable basis for regular follow-up, disease prognosis, and clinical treatment of patients. At present, there are two main methods for lung tumor segmentation one is manual segmentation by doctors, and the other is computer-aided segmentation using segmentation algorithms. Manual segmentation requires doctors with rich experience and a higher professional level. Because manual segmentation has strong subjectivity, the results of segmentation of the same tumor on CT images by doctors with different experiences will also be different. With the implementation and development of precision medicine, computer-aided medical image segmentation technology will face greater challenges [6,7]. Although automatic and semi-automatic segmentation algorithms can eliminate the subjective impact of manual segmentation, how to improve the performance of segmentation algorithms to obtain higher segmentation accuracy is still a challenging task.

The segmentation model based on artificial intelligence needs a large number of labeled datasets, and it is often unable to obtain a well-trained model due to the limitation of data volume [8,9,10]. The automatic segmentation method is popular because of its wide application, which includes knowledge-guided methods and information-based methods [11,12]. In some hybrid methods [12,13,14], different algorithms are combined to obtain better segmentation performance, but this seems to increase the complexity of the method. In the process of lesion segmentation, the segmentation performance of Fuzzy C-mean (FCM) will be affected by noise and clutters [15]. Afshar et al. [16] showed that k-means clustering (K-means) has better segmentation performance than FCM in lung tumor segmentation tasks. Active contour algorithm needs initialization definition, and it has a high time cost [17,18]. Statistical region merging (SRM) will show a suitable segmentation effect after finding the best model over some time, but there may be a certain degree of over-segmentation when the local information is very close [19,20].

Our goal is to segment lung tumors automatically and accurately in CT images. The plan is to propose an automatic segmentation method for lung tumors based on an improved region growing algorithm, which can achieve accurate segmentation while eliminating much manual input and interaction. The purpose of this study is to identify and locate lung tumors from CT images using the prior information on lung tumors and then to construct an automatic segmentation method for lung tumors by combining the improved region growing algorithm. In particular, the technical novelty of this study includes the following:

(1) Combining the prior information of lung tumor with the maximum inter-class variance method (OTSU) algorithm, the method of lung tumor location is constructed, and then the automatic selection of initial seed points of region growing algorithm is realized;
(2) After the seed point expansion, the growth restriction conditions and the automatic updating mechanism of the threshold are established. Finally, the combination result of multi-point growth is taken as the final segmentation result;
(3) The segmentation performance of our proposed method is better than the current popular segmentation algorithm.

## 2. Related Work

Dlamini et al. [21] developed a fully automatic lung tumor segmentation and reconstruction system. The segmentation module of the system includes a region-based active contour algorithm and a k-means algorithm. The system automatically performs 3D reconstruction after extracting the tumor contour, which can help doctors with preoperative planning. Gan et al. [22] proposed an automatic segmentation network model based on deep learning, which is composed of 2D convolutional neural networks (CNN) and 3D CNN and can accurately and automatically segment lung tumors from CT images. Wang et al. [23] proposed a deep network model for lung tumor segmentation, which can effectively and automatically segment various complex lung tumors from CT images, especially those with blurred boundaries. Jiang et al. [24] proposed a multi-scale CNN method, which is composed of incremental multiple resolution residually connected network formula and dense multiple resolutions residually connected network formula and can automatically recognize and segment lung tumors from CT images. Zhang et al. [25] proposed an improved 3D dense connected UNet, which can segment lung tumors of an arbitrary shape from CT images. Cui et al. [26] proposed a segmentation method based on a ‘topo-poly’ graph, which is composed of an intensity graph and a topology graph and can accurately segment lung tumors. Afshar et al. [16] proposed a semi-automatic lung tumor segmentation method, which used Gustafson–Kessel clustering for tumor segmentation after using snake optimization to separate the lung from the background. Anshad et al. [27] proposed a tumor segmentation method based on a modified region growth algorithm. After preprocessing the X-ray image, this method uses the prior information on tumor and region growth to achieve automatic tumor segmentation. It is worth noting that this research does not introduce the automatic image binarization process, so this method should be summarized as a semi-automatic segmentation method. Table 1 shows the details of existing studies on tumor segmentation methods.

Although the method based on artificial intelligence can realize automatic segmentation of lung tumors, the segmentation accuracy is not ideal because of the mismatch between the amount of applicable image data and the model. Although the traditional segmentation algorithm can obtain better accuracy in lung tumor segmentation, its disadvantage is that it can not achieve automatic segmentation and depends on manual interaction. It is worth noting that some hybrid segmentation algorithms also increase the complexity of the method. In conclusion, the methods proposed in the existing studies have some limitations in different aspects.

## 3. Materials and Methods

In order to solve the limitations of existing methods that have been found, this section will propose an automatic segmentation method for lung tumors based on an improved region growing algorithm.

### 3.1. CT Data Preprocess

**Figure 1:**
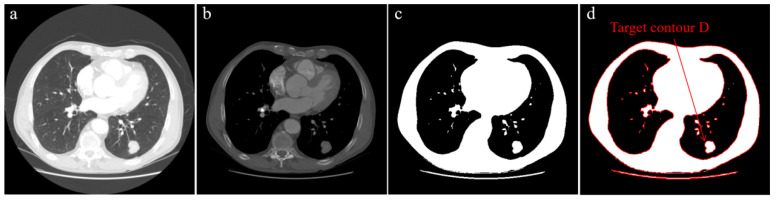
Automatic selection of initial seed point, (**a**) input image, (**b**) preprocessed image, (**c**) binary image, (**d**) after extracting all contours, find the contour that conforms to the attribute and set it as target contour *D*, with its centroid as the initial seed point p1.

We used data from the publicly available LIDC-IDRI dataset, which is provided by https://www.cancerimagingarchive.net/ (accessed on 17 March 2022). The CT dataset of ten patients was selected, and the CT slice size was 512 × 512. As an input image, a CT slice can improve image quality by preprocessing. Adjust the contrast of the input image, which can better display the tumor and can also eliminate the white spots in the input image that will be misdiagnosed as tumors. The preprocessed image is shown in Figure 1b. It is worth noting that image preprocessing is only used as a prerequisite for finding the tumor contour, although it will have some impact on the tumor edge. This is because the segmentation performed by the improved algorithm is before this step.

### 3.2. Region Growing Algorithm

The basic steps of lung tumor segmentation using region growing algorithm are as follows:

(1) Import a CT slice of the lung with the tumor and serve as an input image;
(2) The user manually determines a point on the tumor as the initial seed point;
(3) The color intensity of the initial seed point is saved as a base value;
(4) Set the threshold;
(5) Similarity check;
(6) The adjacent pixels meeting the conditions are added according to the growth rules and saved as growth points;
(7) Check the new adjacent pixels again, add the adjacent pixels that meet the conditions, and save them as growth points;
(8) Until there are no new growth points, the array of obtained pixel points is the segmented tumor area, and the outermost pixels are the segmented tumor boundary.

### 3.3. Improved Region Growing Algorithm

The region growth algorithm is a semi-automatic segmentation method, and its initial seed point and threshold need to be selected and set by manual interaction. In order to solve these problems and optimize the segmentation performance, this section will introduce the workflow of the proposed method in detail.

#### 3.3.1. Automatic Selection of Initial Seed Point

We use the prior information on lung tumors to determine the initial seed point of the improved region growing algorithm, so users do not need to specify the initial seed point. Yu et al. [28] showed that lobectomy is a recommended treatment standard for tumors with a size of 21–30 mm. In the latest international tumor, node, and metastasis (TNM) staging standard for lung cancer (8th edition) [29], the size range of the T1c stage is (>2 to ≤3 cm). According to the size cutting points from T1c to T2b, the size range of lung tumors from T1c to T2b is (>2 to ≤5 cm). The initial seed point of the improved region growing algorithm can be automatically determined by making full use of this information. The specific steps are as follows:

(1) After preprocessing the input image, the binary image is automatically obtained using the OTSU algorithm [30], as shown in Figure 1c;

The OTSU algorithm can automatically calculate the optimal segmentation threshold for the target image. With this optimal segmentation threshold, the target image can be divided into foreground and background images. Therefore, using the OTSU algorithm can automatically realize target image binarization.

(2) Extract all contours in the binary image and draw them, as shown in Figure 1d;
(3) Calculate the area and maximum size of all contours, find the contour that conforms to the attribute and set it as target contour *D*;

The attributes of contour include area and maximum size, so we set two conditions. Where condition a is the limit on the maximum size of the contour, which can be expressed as 2 cm < maximum size ≤ 5 cm. Condition b is the limit on the area of the contour, which can be expressed as 1 cm^2^ < area ≤ 6.25π cm^2^. In condition b, 1 cm^2^ is to eliminate the possible effect of the vessel tree contour, and 6.25π cm^2^ is the area of the circle obtained with a diameter of 5. The contour satisfying both conditions a and b is the target contour *D*.

(4) Find the centroid C of target contour *D* by Equation (1), and set the centroid C as the initial seed point.

(1) $$C=(\frac{\iint_{D}xdxdy}{A},\frac{\iint_{D}ydxdy}{A})$$

Among them, $\frac{\iint_{D}xdxdy}{A}$ is the abscissa, $\frac{\iint_{D}ydxdy}{A}$ is the ordinate, and *A* is the area of target contour *D*.

#### 3.3.2. Initial Seed Point Expansion

The initial seed point is expanded, which actually adopts the ensemble segmentation method [31]. Gu et al. [32] have also proved that the combination of multiple segmentation results of different initialization for multiple regions using the same technology can provide more accurate segmentation results.

**Figure 2:**
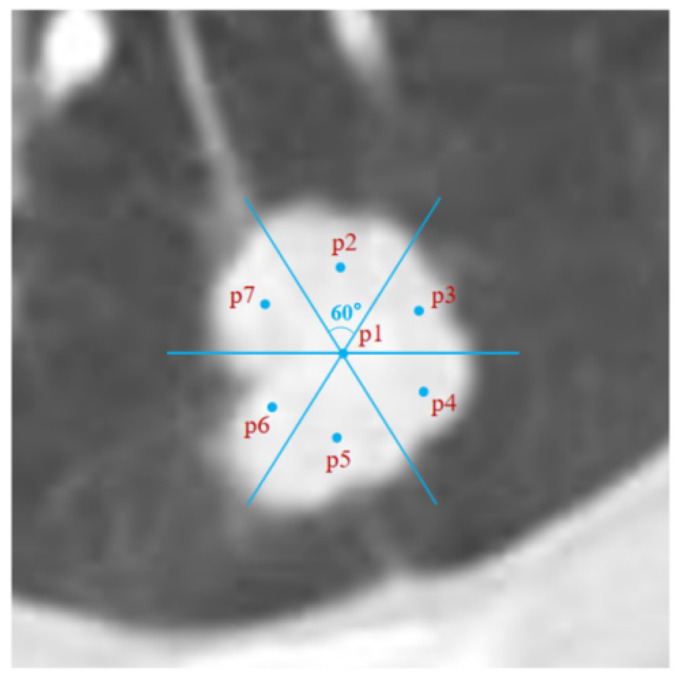
P1 is the initial seed point automatically selected on the target tumor; p1 expands p2, p3, p4, p5, p6, and p7 in a set way and serves as six initial seed points of expansion in the input image.

After the centroid C is determined, make three intersecting dividing lines with centroid C as the origin. All included angles of these dividing lines are 60°, and they are divided into 6 areas. The centroids of these six areas are set as six additional points, namely p2, p3, p4, p5, p6, and p7. If a centroid appears outside the contour of the area, set the midpoint of the minimum distance line between the centroid C and the contour of the area as the expansion point. Mark the positions of these six points in the input image as the six initial seed points of expansion. Figure 2 shows this process. Therefore, the improved region growing algorithm is to queue up from seven initial seed points for region growth, and the final segmentation result is also the combination of the seven segmentation results.

#### 3.3.3. Restrictions

Before lung tumor segmentation, the maximum tumor size can be predicted and determined. In addition, according to the target contour *D* obtained in Section 3.3.1, a rough maximum tumor size can also be obtained. Based on this information, the growth restriction conditions of region growth can be established. If the maximum size of the region growth result exceeds the input predicted maximum size, it is determined as an abnormal result; otherwise, it is a credible result. If the expected maximum size is not input, the longest distance from the centroid C to the target contour *D* is taken as the radius to make a circle. If the area of the region growth result is larger than the area of the circle, it is determined as an abnormal result; otherwise, it is a credible result.

#### 3.3.4. Threshold Detection and Update

In the process of lung tumor segmentation, the threshold value in the region growing algorithm is set as the 20% gray threshold of the input image, which is obtained according to the experience of lung tumor segmentation. When the initial seed point p1 is used for region growth, the threshold value is set to 20% of the gray threshold of the input image for region growth. When the first tumor boundary is obtained, a circle with radius L is made with the previous point (p1) as the center, and 20% of the gray threshold in the circle is used as the threshold value when the p2 initial seed point is used for region growth. L is equal to the maximum distance from the p1 point to the obtained tumor boundary plus 6 units. When the subsequent p3, p4, p5, p6, and p7 points are used for region growth, the updated threshold values are obtained in this way.

#### 3.3.5. Automatic Segmentation Steps

The steps of lung tumor automatic segmentation based on an improved region growing algorithm are as follows:

(1) Import a CT slice of the lung with the tumor and serve as an input image;
(2) Preprocess the input image;
(3) The binary image is obtained by using the OTSU algorithm;
(4) The initial seed point is automatically obtained;
(5) Initial seed point expansion;
(6) Set the threshold value as the 20% gray threshold of the input image;
(7) The color intensity of the initial seed point is saved as a base value;
(8) Similarity check;
(9) The adjacent pixels meeting the conditions are added according to the growth rules and saved as growth points;
(10) Check the new adjacent pixels again, add the adjacent pixels that meet the conditions, and save them as growth points;
(11) Until there are no new growth points, the array of obtained pixel points is the segmented tumor area, and the outermost pixels are the segmented tumor boundary;
(12) Automatically update thresholds;
(13) Repeat steps (7) to (11) with the new threshold obtained, and finally obtain seven tumor boundaries from p1 to p7 of region growth;
(14) According to the growth restriction conditions, the combination of all credible segmentation results obtained from p1–p7 is selected as the final segmentation result.

**Figure 3:**
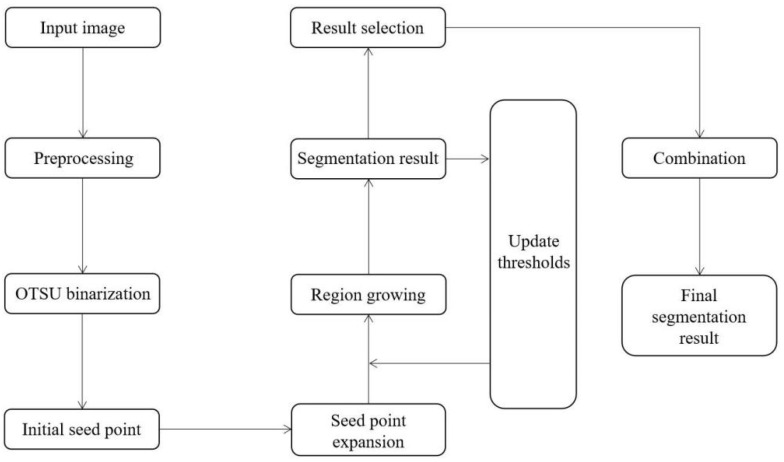
Workflow diagram of our proposed method.

Finally, a lung tumor surface is obtained based on the improved region growing algorithm. Figure 3 shows a workflow diagram of our proposed approach.

## 4. Experiments and Validation

In order to verify the segmentation performance of our proposed method, this section will test the proposed method through 10 groups of experiments and compare it with the current popular segmentation algorithm in the same experimental environment.

### 4.1. Performance Evaluation Metrics

In order to verify the performance of our proposed method, we applied the widely used dice coefficient and Jaccard distance segmentation evaluation metrics to evaluate the segmentation results of our proposed method. These metrics are defined as follows:

(2) $$\mathrm{Dice}\;\mathrm{Coefficients}=\frac{2\left|x\cap y\right|}{\left|x\right|+\left|y\right|}$$

(3) $$\mathrm{Jaccard}\;\mathrm{Distance}=d_{J}(x,y)=1-J(x,y)=\frac{\left|x\cup y\right|-\left|x\cap y\right|}{\left|x\cup y\right|}$$

where *x* is the target tissue pixel points array in the label image, and *y* is the target tissue pixel points array segmented by the test method.

### 4.2. Results and Performance Comparison

CT images from 10 patients in LIDC-IDRI dataset are used as input images. We compare the proposed method with the current popular FCM, K-means, SRM, and active contour algorithms. In these experiments, we mainly focus on the segmentation results and the segmentation performance of our proposed methods. The 10 groups of comparative experiments we designed were implemented using Matlab2016a and were all completed on a single Intel (R) Core (TM) i7-8750H 2.2 GHz/16 GB and NVIDIA geforce GTX 1050Ti computer.

**Figure 4:**
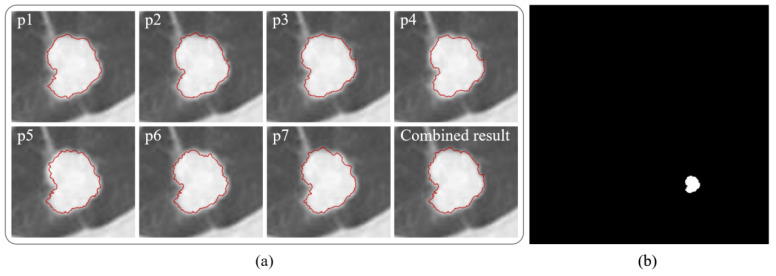
The segmentation result of our proposed method, and the red circle is the tumor boundary obtained after segmentation, (**a**) the target tissue contour obtained from the initial seed points of p1, p2, p3, p4, p5, p6, and p7, respectively, and their combination results, (**b**) final segmentation result.

A sample of the segmentation results of our proposed method is shown in Figure 3. Figure 4a shows the results obtained from the initial seed points of p1, p2, p3, p4, p5, p6, and p7, as well as their combined results. Figure 4b shows the final segmentation results.

**Figure 5:**
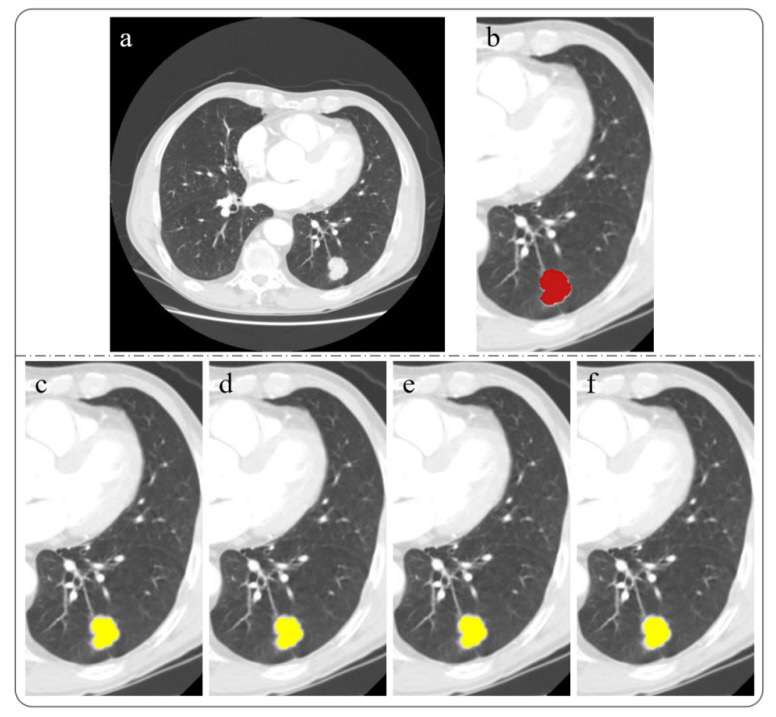
Segmentation results obtained by different methods, the red area is the segmentation result of our method, and the yellow area is the segmentation result of the other four methods, (**a**) input image, (**b**) our method segmentation result, (**c**) FCM segmentation result, (**d**) K-means segmentation result, (**e**) SRM segmentation result, (**f**) active contour segmentation result.

Figure 5 shows the different segmentation results obtained by our proposed method and the current popular FCM, K-means, SRM, and active contour algorithms.

Table 2 shows the dice coefficient and Jaccard distance of our proposed method and other methods in 10 groups of experiments.

**Figure 6:**
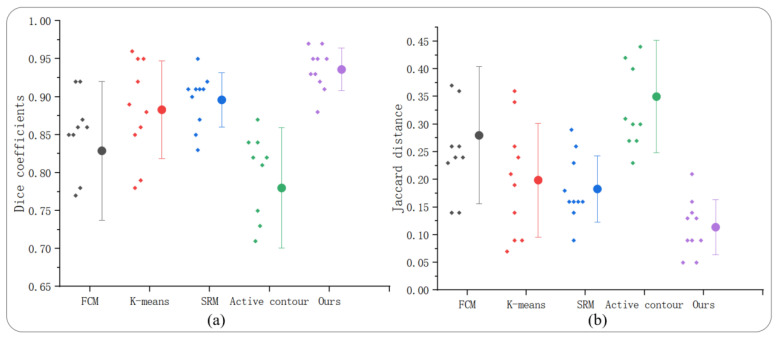
In the LIDC-IDRI dataset, the segmentation performance of our proposed method and the other four methods, (**a**) average dice coefficient, (**b**) average Jaccard distance.

Figure 6 shows the average and standard deviation of the dice coefficient and Jaccard distance obtained by different methods.

**Figure 7:**
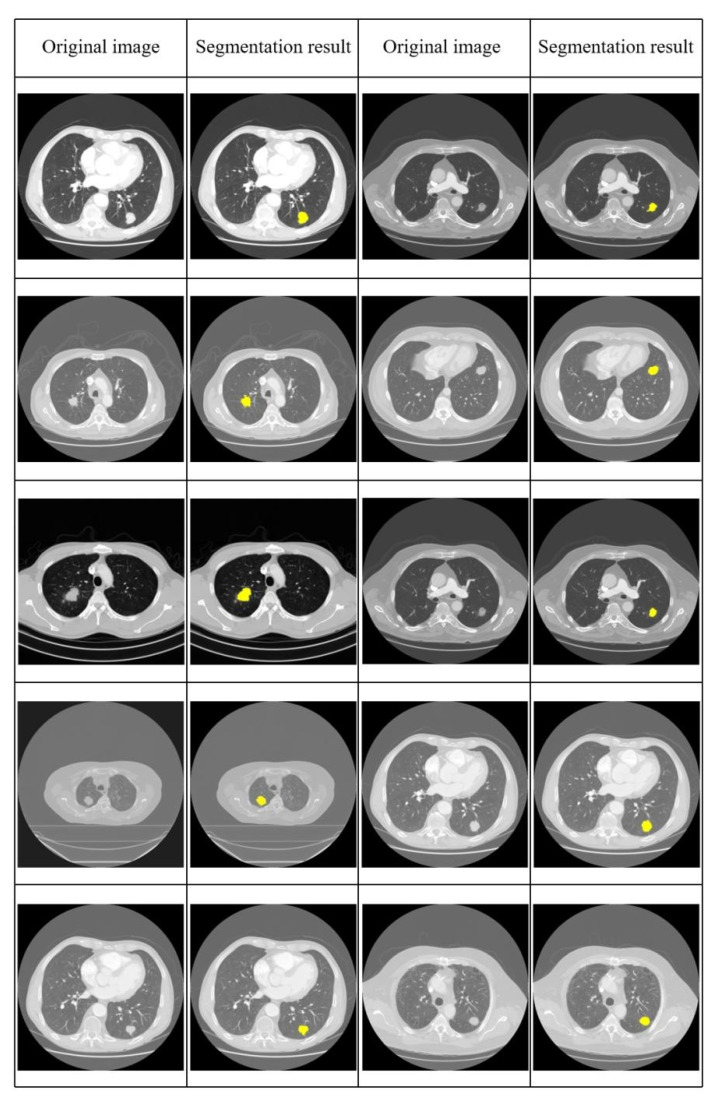
All used image segmentation results obtained with our proposed method, the yellow area represents the segmentation results.

Figure 7 shows all the used image segmentation results obtained with our proposed method.

## 5. Discussion

Ensemble segmentation has a great impact on the performance and segmentation accuracy of the segmentation algorithm, and this study also proves this once again. The segmentation results are mainly affected by two factors. One is the selection of segmentation methods. The other is the adjustment of segmentation methods in specific applications. It can be seen from Table 2 that eight groups of experiments in the LIDC-IDRI dataset of our proposed method have obtained the highest dice coefficient, and one of them is similar to SRM and K-means. We analyzed 10 groups of experimental results. The average dice coefficients obtained by five different methods are 0.829, 0.883, 0.896, 0.780, and 0.936, respectively, and the average Jaccard distance is 0.280, 0.199, 0.183, 0.350, and 0.114, respectively. It can be seen from Figure 6a that the average dice coefficient obtained by our proposed method is 0.936 ± 0.027, which is significantly higher than that of FCM, K-means, SRM, and active contour algorithms. It can be seen from Figure 6b that the average Jaccard distance obtained by our proposed method is 0.114 ± 0.049, which is the best. This means that the segmentation result of our proposed method is closer to manual expert segmentation, which can also indicate that the pixel points not recognized by this method are lower than those of other methods in lung tumor segmentation. In addition, we also compare the segmentation performance of the proposed method with previous research in the same problem domain. Table 3 shows that the segmentation performance of the proposed method is better.

In these experiments, the proposed method did not find obvious over-segmentation, which may be due to the established growth restriction conditions. In the existing studies [11,32,33], lung tumor segmentation restriction that depends on the lung segmentation quality is usually used. It is worth noting that this restriction is likely to exclude the target tumor in the lung segmentation process, and the segmentation failure will occur at this time. However, the growth restriction conditions we established eliminate this problem. Please note that the shape of lung tumors in CT slices of different patients is different, but this shape difference will not affect the performance of the proposed method. Because in the design process of the proposed method, special shapes that may appear in lung tumors, such as concave shapes, have been considered. In Section 3.3.2, the designed solution has excluded the impact of this special shape on the performance of the method.

## 6. Conclusions

We propose an automatic segmentation method for lung tumors based on an improved region growing algorithm, which eliminates much manual interaction and also shows suitable segmentation performance. Experimental results show that the proposed method outperforms the other four popular segmentation methods. The suitable segmentation performance of this method depends on three conditions: the first is the seed point expansion based on ensemble segmentation, the second is the automatic updating mechanism of threshold, and the third is the growth restriction conditions. Although the proposed method has been successfully applied to LIDC-IDRI dataset, there are still some limitations. The proposed method can not automatically segment smaller lung tumors (size < 2 cm) and larger lung tumors (size > 5 cm) because it cannot automatically and accurately recognize lung tumors of this size. Therefore, if the size of the target tumor exceeds the expected range, the initial seed point can not be automatically obtained through this method. In this case, it is necessary to determine the initial seed point through manual interaction to perform subsequent segmentation tasks. In future work, we will involve more target tissue characteristics to reset the prior information and gradually explore the performance of this method to segment different target tissues on other datasets.

## Figures and Tables

**Table 1:** Details of existing studies on tumor segmentation methods.

Study	Method	Dataset	Automation
Afshar et al. [16]	Snake algorithm + Gustafson–Kessel clustering	Unpublished	Semi-automatic
Dlamini et al. [21]	Region-based active contour	LICD-IDRI	Automatic
Gan et al. [22]	Hybrid convolution neural network	Unpublished	Automatic
Wang et al. [23]	Deep learning	Unpublished	Automatic
Jiang et al. [24]	Deep learning	LICD-IDRI + NSCLC	Automatic
Zhang et al. [25]	Deep learning	LICD-IDRI	Automatic
Cui et al. [26]	Topo-poly graph model	NSCLC	Semi-automatic
Anshad et al. [27]	Modified region growing algorithm	Unpublished	Semi-automatic

**Table 2:** In the LIDC-IDRI dataset, the dice coefficients and Jaccard distance obtained by different methods are statistically compared.

Dice Coefficients						Jaccard Distance					
Images	FCM	K-Means	SRM	Active Contour	Ours	Images	FCM	K-Means	SRM	Active Contour	Ours
1	0.86	0.86	0.87	0.84	0.95	1	0.24	0.24	0.23	0.27	0.09
2	0.61	0.85	0.91	0.61	0.92	2	0.56	0.26	0.16	0.56	0.14
3	0.78	0.79	0.85	0.75	0.93	3	0.36	0.34	0.26	0.40	0.13
4	0.92	0.92	0.91	0.82	0.91	4	0.14	0.14	0.16	0.30	0.16
5	0.85	0.95	0.95	0.81	0.95	5	0.26	0.09	0.09	0.31	0.09
6	0.87	0.88	0.91	0.87	0.97	6	0.23	0.21	0.16	0.23	0.05
7	0.77	0.78	0.83	0.73	0.93	7	0.37	0.36	0.29	0.42	0.13
8	0.85	0.89	0.90	0.71	0.88	8	0.26	0.19	0.18	0.44	0.21
9	0.92	0.96	0.92	0.84	0.97	9	0.14	0.07	0.14	0.27	0.05
10	0.86	0.95	0.91	0.82	0.95	10	0.24	0.09	0.16	0.30	0.09

**Table 3:** Comparison of the segmentation performance of our proposed method with previous studies in the same problem domain.

Study	Dice Coefficient (Average)	Jaccard Distance (Average)
FCM	0.829	0.280
K-means	0.883	0.199
SRM	0.896	0.183
Active contour	0.780	0.350
Dlamini et al. [21]	0.921	-
Gan et al. [22]	0.720	0.420
Wang et al. [23]	0.750	-
Jiang et al. [24]	0.710	-
Zhang et al. [25]	0.831	-
Cui et al. [26]	0.881	-
Anshad et al. [27]	0.912	0.161
Ours	0.936	0.114

## Data Availability

Publicly available datasets were analyzed in this study. These data can be found here: [https://www.cancerimagingarchive.net] (accessed on 3 January 2022).
